# UAV Multispectral Remote Sensing for Rice Leaf Blast Severity Grading Using an Improved 1DCNN-Transformer Ensemble Model

**DOI:** 10.3390/plants15182854

**Published:** 2026-09-18

**Authors:** Xiao Liang, Qingbo Song, Hongli Lian, Bo Pang, Hongze Zhang, Fuxu Guo, Ying Zang, Yingli Cao

**Affiliations:** 1 School of Agriculture, Liaodong University, Dandong 118001, China; liaodongliangxiao@163.com (X.L.); lianhongli@liaodongu.edu.cn (H.L.); 13894100819@163.com (B.P.); 2 School of Information Engineering, Huzhou Normal University, Huzhou 313000, China; 2025388020@stu.huznu.edu.cn; 3 College of Information and Electrical Engineering, Shenyang Agricultural University, Shenyang 110866, China; 2023240169@stu.syau.edu.cn (H.Z.); 2023200008@stu.syau.edu.cn (F.G.); caoyingli@syau.edu.cn (Y.C.)

**Keywords:** rice leaf blast, UAV multispectral remote sensing, canopy segmentation, disease-severity classification, 1DCNN-Transformer, ensemble learning, disease mapping, prescription-map decision support

## Abstract

Rice leaf blast can develop rapidly under field conditions, creating a need for fast, non-destructive, and spatially explicit monitoring. UAV multispectral imagery and synchronized ground disease surveys were collected from artificially induced rice leaf blast experiments conducted in 2024 and 2025. After image registration, U-Net canopy segmentation, and ROI quality screening, 1801 curated 3 × 3-pixel canopy ROIs were retained. Each ROI was represented by four reflectance bands and 10 vegetation indices selected by Pearson correlation analysis, and the resulting dataset supported model development and spatial mapping. The improved 1DCNN-Transformer branch combined multi-scale Inception convolution, SE recalibration, and Focal Loss with RF and GBDT probability fusion. In the hold-out evaluation, the model achieved an overall accuracy of 98.90% and a weighted F1-score of 98.89%. Five repetitions of five-fold grouped cross-validation were performed, with identical 14-feature vectors constrained to the same fold. RF, GBDT, and equal RF-GBDT probability fusion achieved mean accuracies of 99.63%, 99.29%, and 99.33%, respectively, supporting strong class separability after duplicate-group isolation. The resulting severity and prescription maps provide an end-to-end digital workflow from canopy extraction to spatial decision support.

## 1. Introduction

Rice is a staple food crop in China and many other rice-growing regions. Rice blast, caused by Magnaporthe oryzae, can occur throughout crop development and threaten both plant vigor and yield stability [1,2]. Field diagnosis still relies heavily on visual scoring and manual sampling. Although these approaches can be accurate, they are slow, labor-intensive, and spatially limited, reducing their suitability for rapid monitoring across large paddy fields [3,4,5].

UAV remote sensing enables rapid, repeated acquisition of field-scale crop imagery and has become an important tool for disease monitoring. Recent deep-learning studies have addressed fine-grained rice phenotyping, UAV-based rice disease mapping, compact plant-disease detection, and multi-class disease classification [6,7,8,9]. Related work has also examined green-control practices for rice diseases [10], multi-scale convolutional detection of rice leaf blast [11], and the broader use of UAV remote sensing for rice and crop monitoring [12,13]. Across other crop systems, hyperspectral and multispectral imagery has supported chlorophyll estimation, yield prediction, and disease detection [14,15,16,17,18,19]. For rice leaf blast specifically, Zhao et al. [20] integrated UAV remote sensing, a disease-sensitive vegetation index, and machine learning, whereas Liu et al. [21] combined UAV hyperspectral imagery with a multi-scale attention Transformer. Compared with RGB imagery, multispectral imagery captures green, red, red-edge, and near-infrared responses associated with pigments, moisture, tissue structure, and canopy condition. Compared with hyperspectral data, UAV multispectral data are less computationally demanding and more practical for repeated field deployment.

Paddy UAV images usually contain rice canopies, soil, water, ridges, and weeds in the same scene. If spectral features are extracted directly from mixed pixels, background noise can mask the disease signal and reduce model stability. Ronneberger et al. [22] introduced U-Net as an encoder–decoder network for pixel-level semantic segmentation. Cao et al. [23] combined U-Net with SAM-assisted annotation to extract rice fields from remote-sensing images. Ground-truth disease grading followed GB/T 15790-2009, Rules for the Investigation and Forecasting of Rice Blast [24]. To reduce background interference in the present study, U-Net semantic segmentation was used to isolate rice canopy regions and build disease-grading samples from masked multispectral images.

For severity grading, a dual-branch 1DCNN-Transformer structure was used as the baseline. The 1DCNN branch extracts local spectral-combination features, whereas the Transformer branch models global dependencies among bands and vegetation indices. Xue and Su [25] reviewed the development and applications of remote-sensing vegetation indices. Gitelson et al. [26] related leaf chlorophyll content to spectral reflectance for non-destructive assessment. Qiao et al. [27] evaluated vegetation-index responses in UAV-based chlorophyll estimation under different crop coverages. Li et al. [28] showed the usefulness of red-edge spectral vegetation indices for estimating crop nitrogen status. Considering the low-dimensional and structured nature of the input features, the baseline was further improved by adding multi-scale 1D Inception convolution, SE feature recalibration, Focal Loss, Random Forest (RF), Gradient Boosting Decision Trees (GBDT), and validation-set probability-weighted fusion. Fawaz et al. [29] proposed InceptionTime for multi-scale convolutional time-series classification. Hu et al. [30] introduced squeeze-and-excitation networks for adaptive channel recalibration. Lin et al. [31] proposed Focal Loss to emphasize hard-to-classify samples. Breiman [32] established Random Forests as an ensemble of randomized decision trees. Friedman [33] formulated gradient boosting as sequential function approximation. Wolpert [34] introduced stacked generalization for combining the outputs of multiple models.

Despite recent progress, three gaps remain. First, operational management requires canopy-scale severity classification and spatial mapping rather than leaf-level recognition alone. Second, complex paddy backgrounds can contaminate canopy spectra, requiring segmentation, vegetation-index interpretation, and severity modeling to be integrated within a single workflow. Third, field management requires class predictions to be translated into spatially explicit decision-support products. Accordingly, this study developed and evaluated an integrated UAV multispectral workflow for six-class rice leaf blast severity grading, disease mapping, and prescription-map generation. The four main contributions are summarized below.

(1) A two-year canopy-level UAV multispectral dataset was curated from synchronized field ratings, with each modeling sample defined as a 3 × 3-pixel ROI representing a local rice-canopy unit.
(2) A U-Net-based masking workflow was integrated with multispectral preprocessing to reduce soil, water, ridge, and weed interference before spectral feature extraction.
(3) An improved 1DCNN-Transformer deep branch was integrated with RF and GBDT probability outputs, and ablation experiments were conducted to quantify the contributions of the individual model components.
(4) Severity predictions were converted into georeferenced disease-severity and prescription maps, establishing a workflow from canopy-level classification to spatial output generation.

## 2. Materials and Methods

### 2.1. Experimental Area and Artificial Induction Design

**Figure 1:**
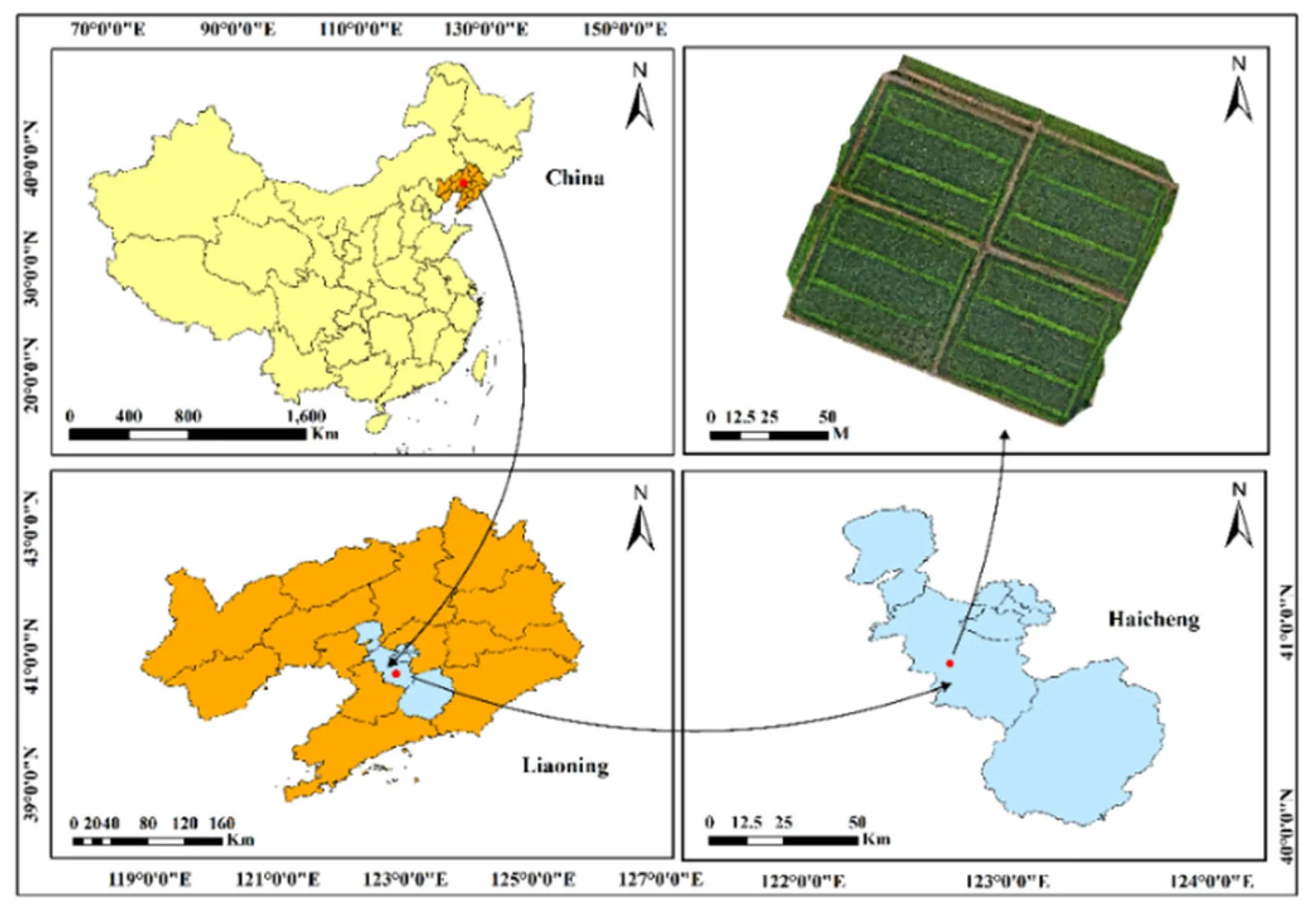
Location and layout of the 16-plot rice leaf blast induction experiment in Haicheng City, Liaoning Province, China; UAV and field surveys were conducted from May to August in 2024 and 2025.

Artificial induction experiments for rice leaf blast were conducted from May to August in both 2024 and 2025 at the rice experimental base of Shenyang Agricultural University in Haicheng City, Liaoning Province, China (Figure 1). The rice cultivar Shennong 9903 was selected because of its relatively low resistance and suitability for disease induction.

A high-density planting pattern was used, with plants spaced 10 cm apart and rows spaced 15 cm apart. Sixteen 2 m × 2 m plots were established in each field season, with at least 5 m between plots. The same experimental protocol and 16-plot layout were used in 2024 and 2025. Samples from both years were pooled for modeling, and year and plot effects were not estimated separately. During the jointing stage, inoculation was performed on humid, cloudy evenings. Leaves were lightly punctured, sprayed with a rice blast spore suspension, and maintained under humid conditions to promote consistent disease development.

### 2.2. Data Acquisition

#### 2.2.1. UAV Multispectral Data Acquisition

**Figure 2:**
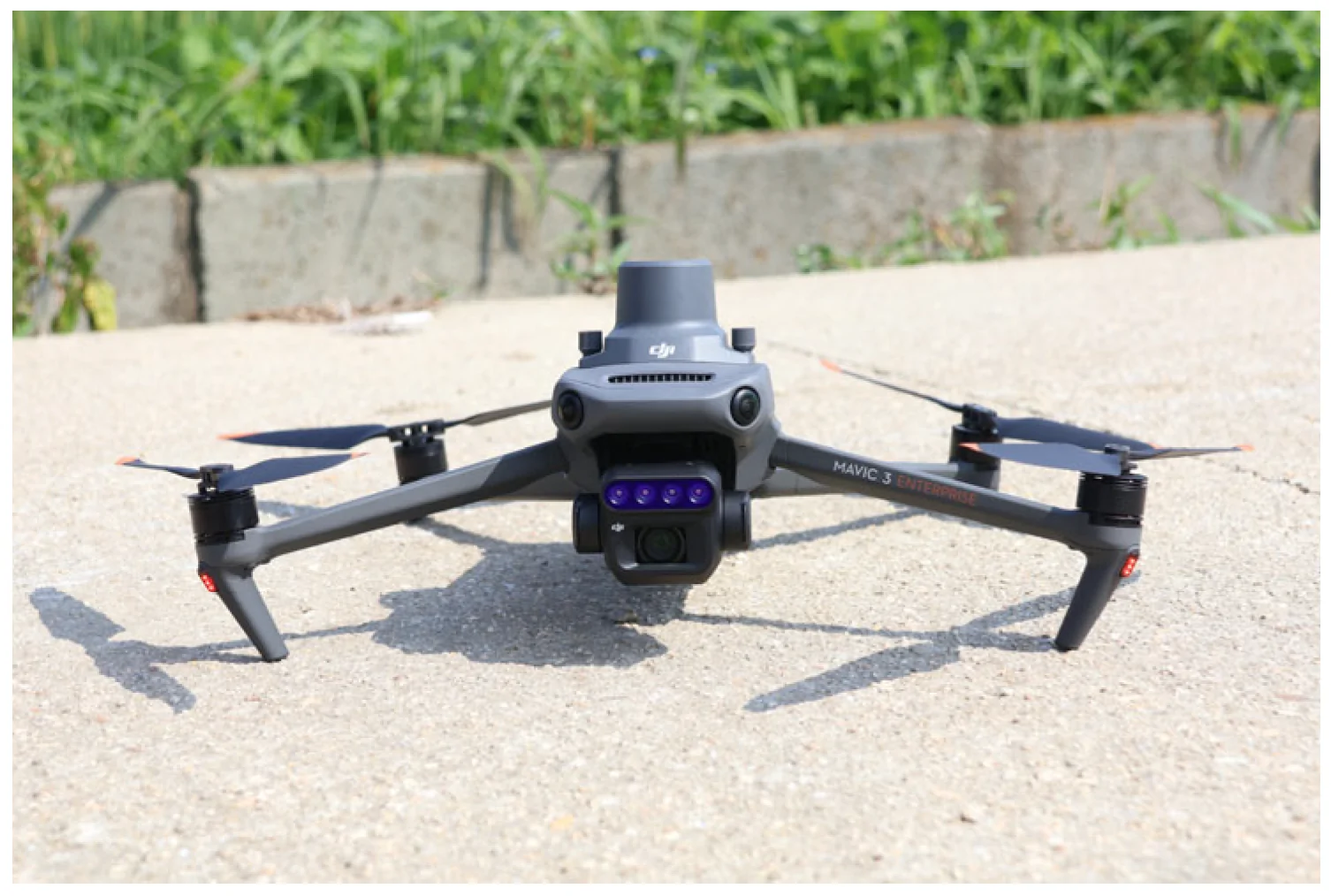
DJI Mavic 3 Multispectral UAV used to acquire green, red, red-edge, and near-infrared rice-canopy imagery under the flight conditions described in Section 2.2.1.

Rice-canopy multispectral images were acquired using a DJI Mavic 3 Multispectral UAV (SZ DJI Technology Co., Ltd., Shenzhen, China) (Figure 2). Flights were conducted under stable illumination, low wind, no precipitation, and no obvious cloud-shadow interference, mainly between 10:00 and 14:00. The flight altitude was 30 m, with 80% forward overlap and 70% side overlap. Flight planning and orthomosaic and radiometric preprocessing were performed in DJI Terra (version 3.8.1) (SZ DJI Technology Co., Ltd., Shenzhen, China). The principal sensor and acquisition parameters are summarized in Table 1.

#### 2.2.2. Rice Leaf Blast Severity Survey

Disease severity was investigated according to GB/T 15790-2009, Rules for the Investigation and Forecasting of Rice Blast [24]. Before each UAV flight, five sampling points were randomly selected in each plot, and 50 rice plants were investigated at each point. Individual leaves were assigned the standard field ratings 0, 1, 3, 5, 7, or 9 according to lesion number, lesion distribution, and diseased leaf-area ratio. Investigators were trained before field scoring to keep the criteria consistent. These field ratings are intermediate inputs to the plot-scale disease-index calculation and are not the same as the six model class labels (Table 2).

For modeling and mapping, canopy-level leaf blast severity was represented by six consecutive classes (0–5): healthy, mild, slightly mild, moderate, moderately severe, and severe. The disease index (DI) was calculated at the plot scale from the standard field ratings as follows: DI = [sum (number of diseased leaves at each rating × representative rating value)/(total investigated leaves × highest rating value)] × 100. The resulting DI was then converted to model classes 0–5 using the thresholds in Table 3. Thus, ratings 0, 1, 3, 5, 7, and 9 describe symptoms on individual leaves, whereas classes 0–5 are canopy-level categories derived from DI. Ground markers were placed during field surveys to spatially match the observations with UAV image regions.

### 2.3. UAV Multispectral Image Preprocessing and Complex Background Removal

The UAV multispectral images were registered in ENVI 5.6 (NV5 Geospatial Solutions, Broomfield, CO, USA) to align the red, green, near-infrared, and red-edge bands at the pixel level. Band synthesis and GeoTIFF export were completed in ArcGIS(Version 10.8, developed by Esri Inc., Redlands, CA, USA). To remove soil, water, ridges, weeds, and other complex backgrounds, the composite images were cut into 256 × 256-pixel patches. Rice-canopy regions were annotated with SAM assistance, and the annotated patches were used to build the U-Net semantic-segmentation dataset [22,23]. Global Mapper (Version 24.0, developed by Blue Marble Geographics, Hallowell, ME, USA) was used for subsequent prescription-map conversion.

The U-Net input channel number was set to four, corresponding to the four multispectral bands, and the output channel number was set to one to distinguish the rice canopy from the background. The dataset was divided into training and validation sets at a 7:3 ratio, with 140 image patches for training and 60 for validation. The validation Dice coefficient was used as the main evaluation metric. After comparing the learning rate, optimizer, batch size, and loss function, the final training configuration was determined as shown in Table 4.

After 300 training epochs, the segmentation model converged. Training loss decreased to approximately 2.38%, whereas the validation Dice coefficient increased and then stabilized. The training and validation trajectories showed no obvious divergence during optimization. The final validation Dice coefficient was 93.36%, indicating substantial overlap between predicted canopy masks and manual labels (Table 5).

### 2.4. Construction of the Multispectral Rice Leaf Blast Grading Dataset

After background removal and mask processing, rice-canopy regions were labeled using the ROI tool in ENVI 5.6. Each sample represented a local canopy unit centered on a labeled rice hill. Reflectance was averaged across a 3 × 3-pixel ROI so that the nine pixels were not treated as independent observations. The workflow retained 1801 high-quality ROI samples across severity classes 0–5 (Table 6). The near-balanced distribution was intentional. Artificial inoculation generated a broad severity gradient, and qualified ROIs were selected within the six predefined disease-index intervals to prevent more common classes from dominating model development. These proportions therefore describe the analytical sampling design rather than the natural prevalence of severity grades in the field.

The final dataset contained 1801 ROI records. For each ROI, four reflectance bands were extracted, and 20 vegetation indices were initially calculated. Pearson correlation analysis was subsequently used to select 10 vegetation indices strongly associated with disease severity. The four spectral bands and the 10 selected vegetation indices were combined to form a 14-predictor feature vector for model training, evaluation, and spatial mapping. Classes 0–5 contained 300, 300, 300, 300, 300, and 301 records, respectively.

### 2.5. Analysis of Vegetation Indices Associated with Rice Leaf Blast Severity

Twenty vegetation indices associated with crop condition and disease response were calculated from the green, red, red-edge, and near-infrared bands. Pearson correlation analysis was used to screen these candidate indices. The 10 indices with |r| > 0.6 were retained and combined with the four original spectral bands, resulting in a 14-predictor feature set used for subsequent model development and spatial mapping (Table 7).

**Table 7:** Twenty vegetation indices calculated from the four Mavic 3M bands for exploratory severity association screening. B1, B2, B3, and B4 denote green, red, red-edge, and near-infrared reflectance, respectively.

Index	Abbreviation	Formula	Reference
Normalized Difference Vegetation Index	NDVI	(B4 − B2)/(B4 + B2)	[35]
Green Normalized Difference Vegetation Index	GNDVI	(B4 − B1)/(B4 + B1)	[36]
Normalized Difference Red Edge Index	NDRE	(B4 − B3)/(B4 + B3)	[28]
Ratio Vegetation Index	RVI	B4/B2	[37]
Red-edge Ratio Vegetation Index	RE-RVI	B4/B3	[38]
Green Chlorophyll Index	GCI	(B4/B1) − 1	[26]
Green-Red Ratio	GR	B1/B2	[25]
Green-Red Difference Index	GMR	B1 − B2	[39]
MERIS Terrestrial Chlorophyll Index	MTCI	(B4 − B3)/(B4 − B2)	[40]
Optimized Soil-Adjusted Vegetation Index	OSAVI	1.16(B4 − B2)/(B4 + B2 + 0.16)	[41]
Triangular Vegetation Index	TVI	60(B4 − B1) − 100(B2 − B1)	[42]
Soil-Adjusted Vegetation Index	SAVI	1.5(B4 − B2)/(B4 + B2 + 0.5)	[43]
Modified Simple Ratio	MSR	(B4/B2 − 1)/(B4/B2 + 1)^0.5^	[44]
Modified Chlorophyll Absorption Ratio Index	MCARI	(B3 − B2) − 0.2(B3 − B1)(B3/B2)	[45]
Leaf Chlorophyll Index	LCI	(B4 − B3)/(B4 + B2)	[46]
Carotenoid Reflectance Index II	CRI2	1/B1 – 1/B4	[47]
Simplified Canopy Chlorophyll Content Index	SCCCI	[(B4 − B3)/(B4 + B3)]/[(B4 − B2)/(B4 + B2)]	[48]
Chlorophyll Vegetation Index	CVI	(B4B2)/B1^2^	[49]
Green Difference Vegetation Index	DVIGRE	B4 − B1	[27]
Red-edge Difference Vegetation Index	DVIRED	B4 − B3	[50]

Note: B1, B2, B3, and B4 represent the green, red, red-edge, and near-infrared bands, respectively.

**Figure 3:**
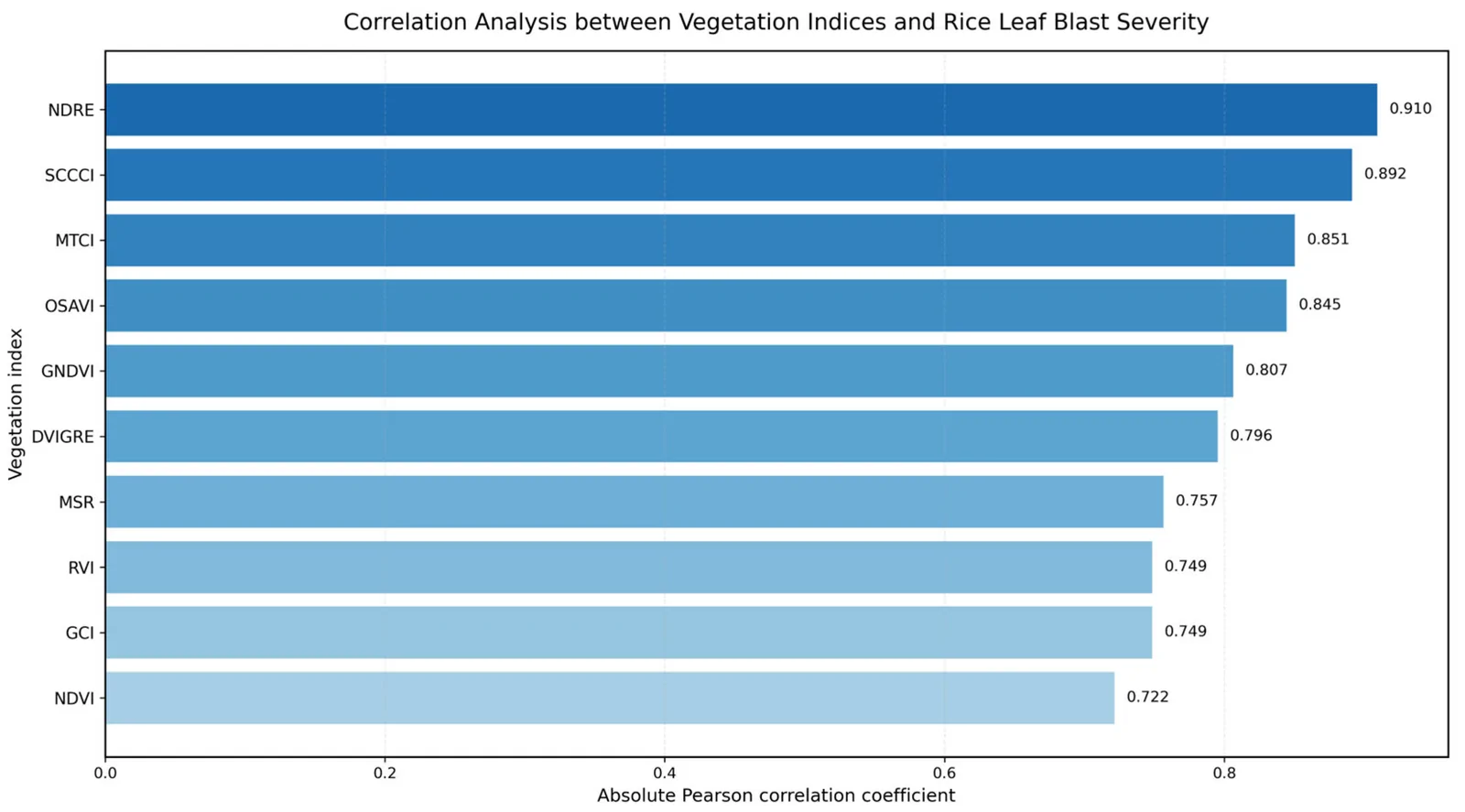
Pearson correlations between the 20 candidate vegetation indices and rice leaf blast severity classes in the 1801 field-derived ROI samples. The 10 indices with |r| > 0.6 were selected for subsequent model development.

Pearson correlation analysis identified NDRE, SCCCI, MTCI, OSAVI, GNDVI, DVIGRE, MSR, RVI, GCI, and NDVI as the 10 indices with |r| > 0.6 (Figure 3). These indices were combined with the four original spectral bands to form the 14-dimensional feature vector used for model training, evaluation, and spatial mapping.

### 2.6. Rice Leaf Blast Severity Classification Using 1DCNN-Transformer and Ensemble Learning

#### 2.6.1. Architecture of the 1DCNN-Transformer Dual-Branch Baseline Model

**Figure 4:**
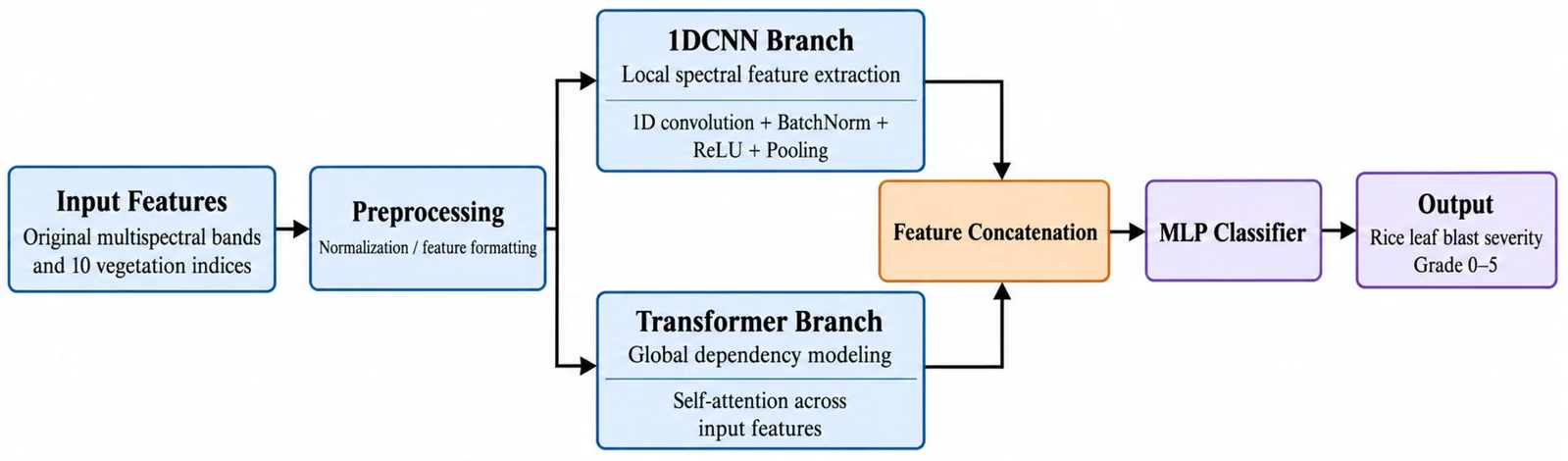
Baseline dual-branch 1DCNN-Transformer architecture for rice leaf blast severity classification.

The baseline used a dual-branch 1DCNN-Transformer architecture (Figure 4). The input layer received a multidimensional feature vector comprising the original multispectral bands and vegetation indices. The 1DCNN branch applied one-dimensional convolution, batch normalization, ReLU activation, and pooling to extract local spectral-combination features, whereas the Transformer branch used self-attention to model global dependencies among the inputs. The branch outputs were concatenated and passed through a multilayer perceptron to predict rice leaf blast severity classes 0–5.

#### 2.6.2. Transformer Mechanism and Multispectral Feature Interaction

The Transformer mechanism is suitable for this task because the multispectral grading features are not isolated variables. Original bands and vegetation indices jointly describe changes in leaf pigment, moisture, tissue structure, and lesion expansion. Compared with convolutional structures that rely mainly on fixed local receptive fields, the Transformer can establish long-range dependencies and global feature interactions, making it useful for capturing the coordinated spectral responses associated with disease severity.

#### 2.6.3. Improvement of the Deep Learning Branch

**Figure 5:**
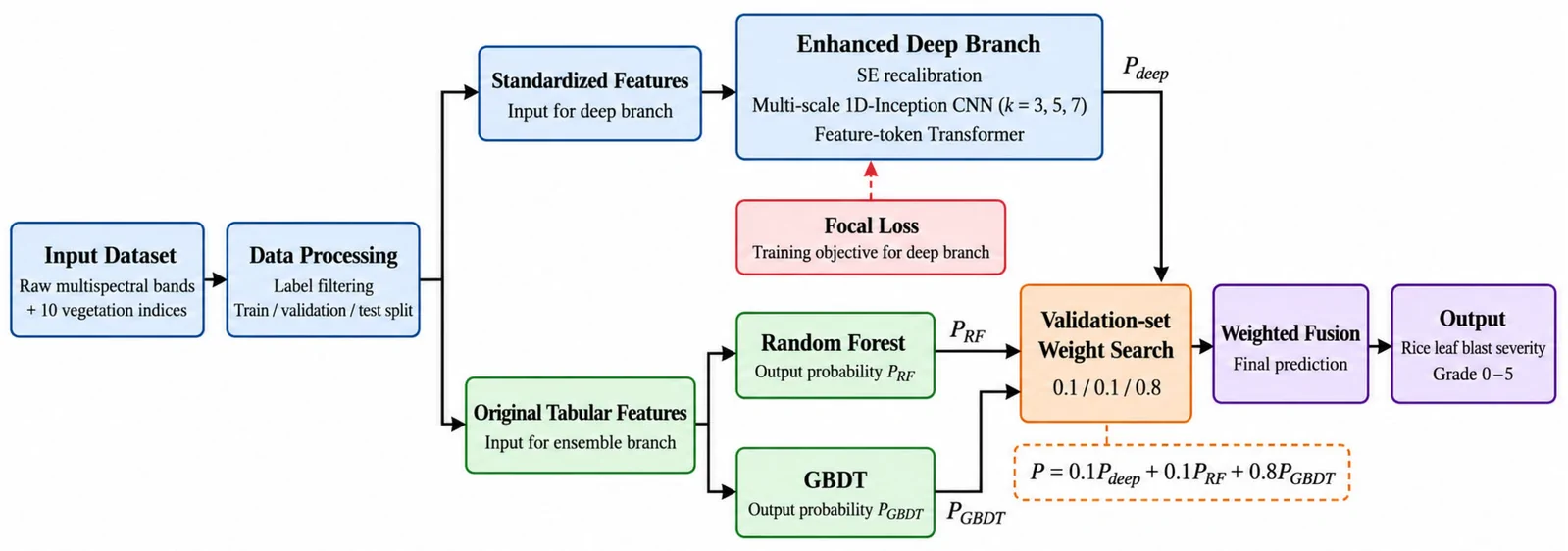
Probability-level ensemble framework combining the improved deep branch, RF, and GBDT; final validation-selected weights were 0.1, 0.1, and 0.8, respectively.

Six methodological changes were introduced to the baseline model (Table 8). Standard one-dimensional convolution was replaced with a multi-scale 1D Inception structure using parallel kernel sizes of 3, 5, and 7. SE recalibration was added at the input-feature and fused-feature levels to assign adaptive weights to informative bands and indices. Focal Loss (gamma = 2.0) was used to down-weight easily classified samples and emphasize difficult observations near adjacent severity boundaries, rather than as a mechanism for correcting class imbalance (Figure 5).

#### 2.6.4. Ensemble Learning Branches and Probability-Weighted Fusion

Because the improved-model dataset comprised low-dimensional, structured spectral and vegetation-index features, RF and GBDT were added as ensemble branches. RF is robust to small sample sizes, nonlinear feature combinations, and redundant variables [32], whereas GBDT sequentially fits residual errors and can represent nonlinear decision boundaries [33]. The deep branch, RF, and GBDT each produced class probabilities for severity classes 0–5. Fusion weights were selected using validation predictions only. Candidate weights ranged from 0.0 to 1.0 in increments of 0.1, summed to 1.0, and assigned at least 0.1 to each included branch. Validation OA was maximized, with weighted F1 used to resolve ties. This procedure yielded P = 0.1 × PDeep + 0.1 × PRF + 0.8 × PGBDT, while the test set was reserved for final hold-out evaluation. Saved-model metadata recorded Lightning 2.6.5 for the deep checkpoint and scikit-learn 1.6.1 for the RF and GBDT files.

#### 2.6.5. Training Configuration, Ablation Experiments, and Evaluation Metrics

The 1801-record ROI dataset was partitioned using StratifiedShuffleSplit (random_state = 42) into 1260 training, 360 validation, and 181 test records. Duplicate-group-aware validation was conducted to assess model robustness under stricter record separation. Records with identical values across all 14 predictors were assigned to the same group, and five-fold StratifiedGroupKFold was repeated five times using random seeds 42, 52, 62, 72, and 82. RF and GBDT were refitted within each training fold using the same 14 predictors. Because tree models do not require standardization, no full-dataset scaling was applied. A pre-specified equal-probability RF-GBDT fusion was also evaluated. OA and weighted F1 were summarized as mean +/– SD across 25 paired folds with descriptive 95% t intervals, and two-sided paired sign tests compared the fusion with each individual tree model.

The grouped analysis ensured that no identical 14-feature group appeared in both the training and test partitions of a fold. Because the dataset did not include plot identifiers, this analysis was defined as duplicate-group-aware validation rather than leave-one-plot-out validation. This procedure therefore assessed independence with respect to exact duplicate feature records, rather than spatial or plot-level independence.

Training used a maximum of 80 epochs, a batch size of 16, a learning rate of 5 × 10^−5^, Focal Loss with gamma = 2.0, early stopping with a patience of 10, and random seed 42. RF used 600 trees, and GBDT used 1800 class-wise boosting trees in the ablation analysis. Because neural-network FLOPs and tree-path operations are not directly comparable, they are reported only as implementation descriptors.

## 3. Results

### 3.1. Complex Background Removal in Paddy Fields

**Figure 6:**
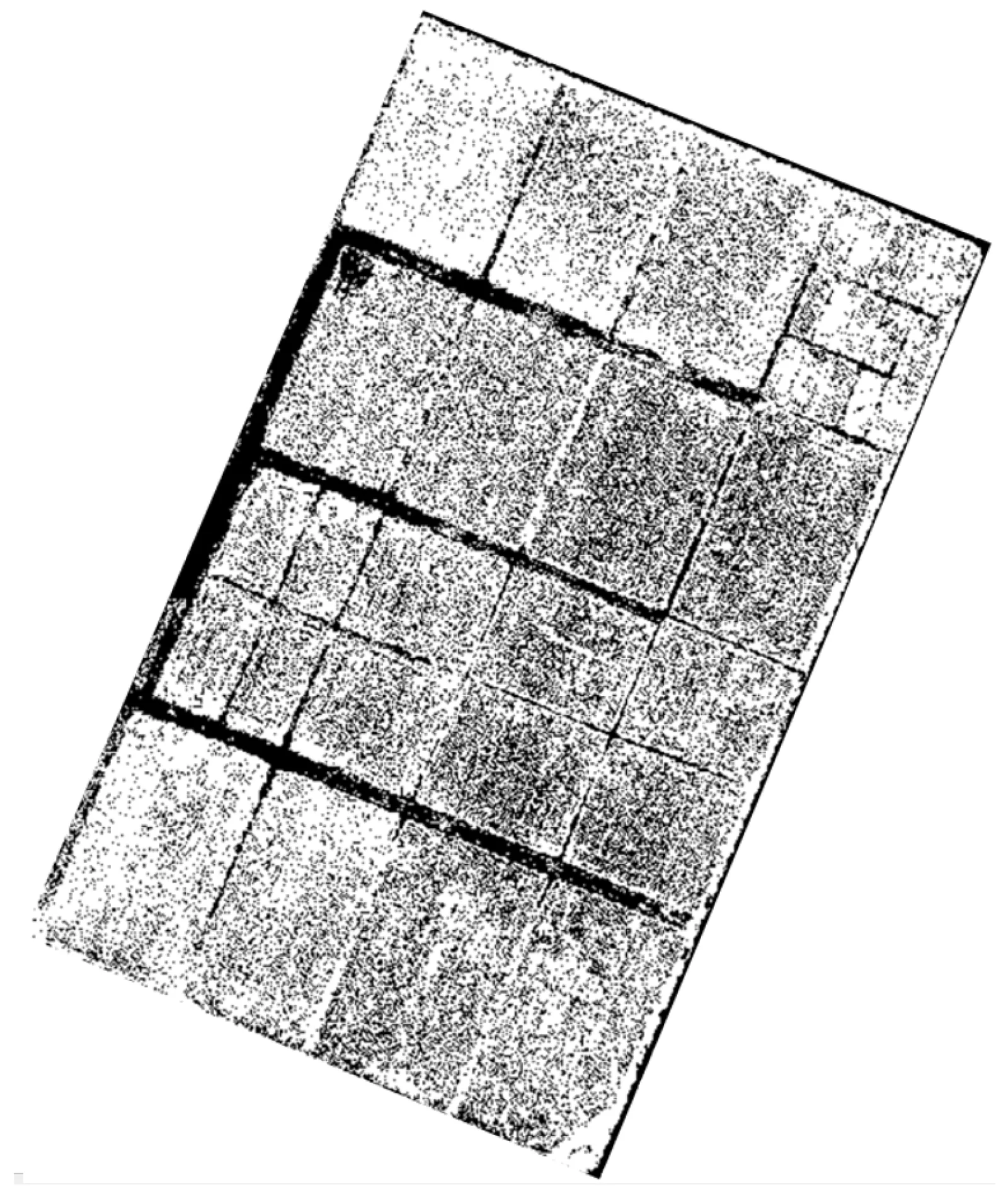
Representative binary rice-canopy mask generated by the U-Net model; white pixels denote canopy and black pixels denote non-canopy background.

After training, the full-field UAV multispectral image was processed by the U-Net model to extract the rice canopy from the background. The registered composite image was used as input, and the model produced a binary mask of the same dimensions. Black pixels represented the background, and white pixels represented the rice canopy (Figure 6).

**Figure 7:**
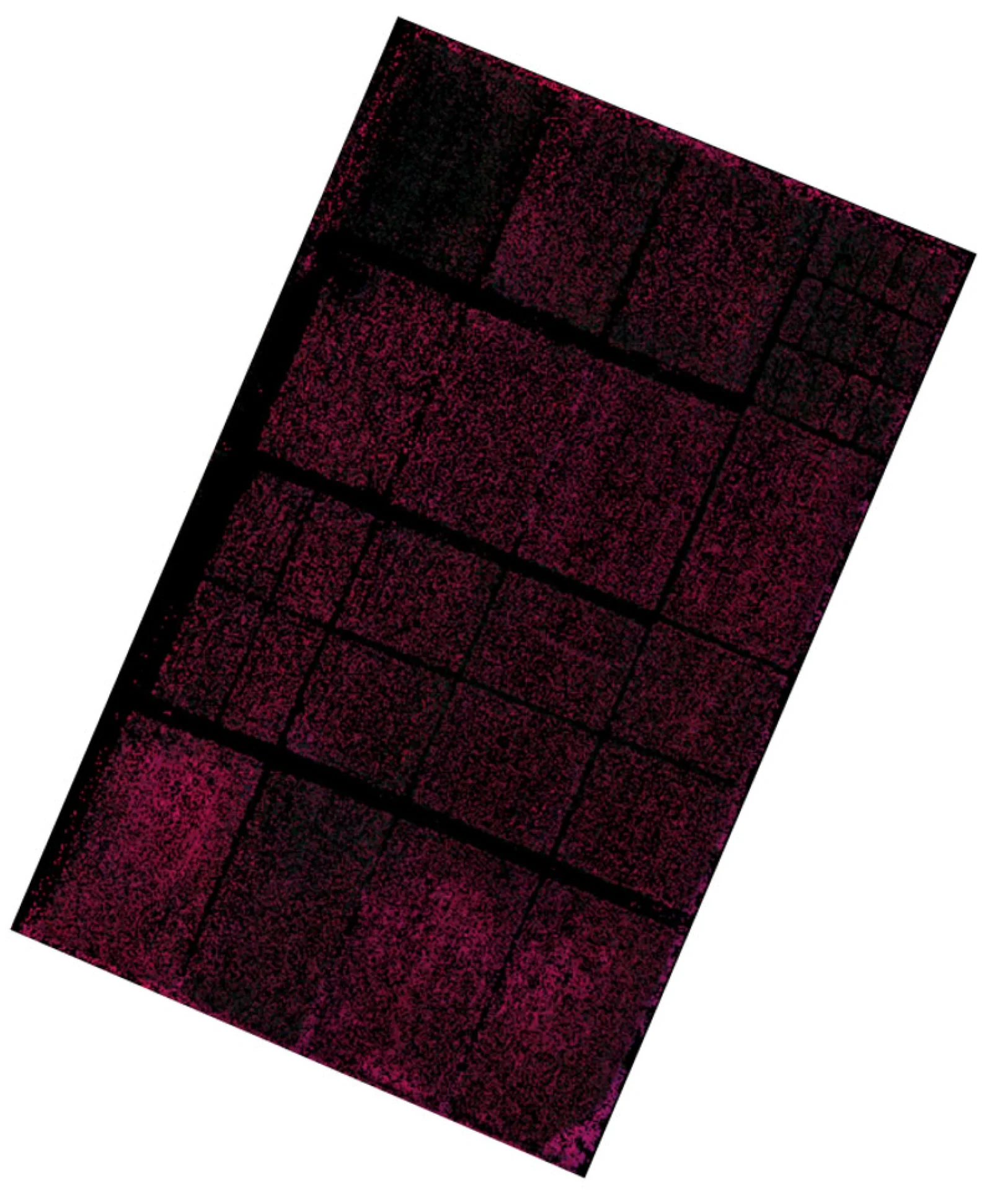
Representative four-band rice-canopy image after multiplying the U-Net binary mask by the registered multispectral image to suppress soil, water, ridge, and weed pixels.

**Figure 8:**
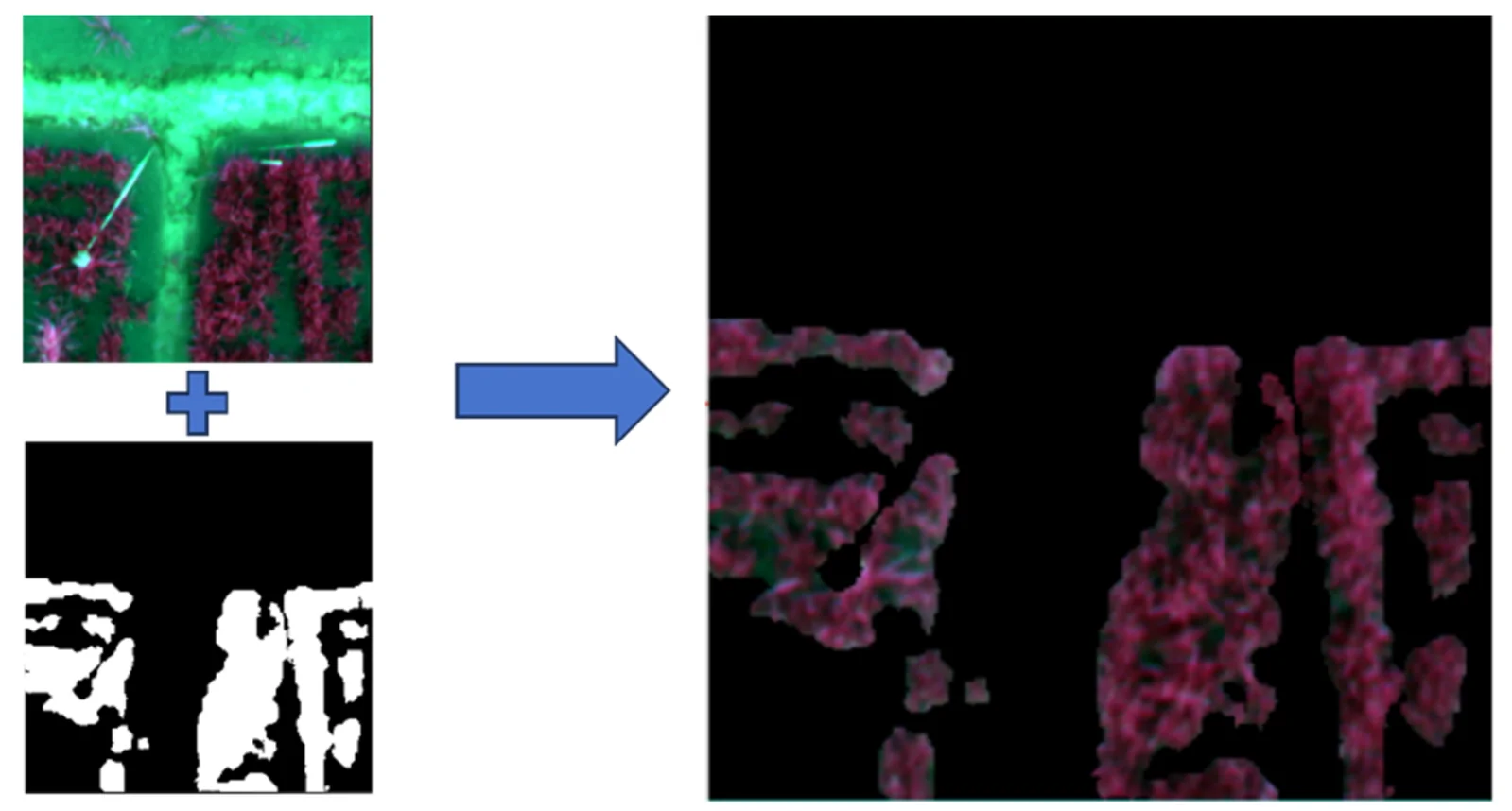
Enlarged example of the masked rice canopy used for 3 × 3-pixel ROI extraction after complex background removal.

The binary mask was multiplied pixel by pixel with the original UAV multispectral image. This operation retained spectral information from canopy pixels and set background pixels to zero, producing a masked rice-canopy image (Figure 7). Visual inspection indicated that the masked image preserved canopy boundaries and internal structure; a representative local example is shown in Figure 8.

### 3.2. Multispectral Grading Dataset and Vegetation-Index Screening

The field-derived dataset comprised 1801 curated ROI samples, with 300–301 samples per class (16.66–16.71%; Table 6). This near-balanced distribution resulted from targeted sampling within the predefined disease-index intervals and subsequent quality screening. Twenty candidate vegetation indices were initially calculated, of which 10 indices with |r| > 0.6 were selected and combined with the four spectral bands to yield 14 predictors per ROI. The same 14-predictor feature set was used for model evaluation and spatial mapping. The balanced design enabled all six severity classes to be evaluated without majority-class dominance.

### 3.3. Model Performance and Ablation Results

As shown in Table 9, the baseline 1DCNN-Transformer achieved an OA and weighted F1-score of 96.13% on the test set. Adding the improved deep branch produced a modest increase in Precision, while the remaining metrics were nearly unchanged. Thus, improving the deep branch alone provided limited benefit for the structured feature set used here.

As shown in Table 10, under the hold-out split, the full three-branch model correctly classified 179 of 181 records, corresponding to an OA of 98.90%, weighted Precision of 98.93%, weighted Recall of 98.90%, and weighted F1-score of 98.89%. The descriptive 95% Wilson interval for OA was 96.06–99.70%. RF and GBDT were further evaluated using repeated duplicate-group-aware validation.

Across 25 duplicate-group-aware test folds, RF achieved an OA of 99.63% +/– 0.26% (95% CI: 99.52–99.74%) and a weighted F1-score of 99.63% +/– 0.26%. GBDT achieved 99.29% +/– 0.46% OA and 99.29% +/– 0.46% weighted F1, whereas equal RF-GBDT probability fusion achieved 99.33% +/– 0.43% for both metrics (Table 11). The fusion did not differ significantly from GBDT in the paired sign test (OA and F1, *p* = 0.125) and performed below RF (OA *p* = 0.00027; F1 *p* = 0.00040). All three approaches nevertheless remained above 99% after identical feature records were confined to the same fold, supporting the conclusion that duplicate records alone did not account for the high classification performance.

**Table 10:** Component and probability-fusion ablation results under the 1801-record hold-out split. Fusion weights were selected using validation data.

Component	Deep Weight	RF Weight	GBDT Weight	Validation OA	Test OA	Test F1	Correct Samples
Deep	1.00	0.00	0.00	98.89%	96.13%	96.19%	174/181
RF	0.00	1.00	0.00	99.72%	98.90%	98.89%	179/181
GBDT	0.00	0.00	1.00	99.72%	98.90%	98.89%	179/181
Deep + RF	0.10	0.90	0.00	99.72%	98.90%	98.89%	179/181
Deep + GBDT	0.10	0.00	0.90	99.72%	98.90%	98.89%	179/181
RF + GBDT	0.00	0.10	0.90	99.72%	98.90%	98.89%	179/181
Deep + RF + GBDT	0.10	0.10	0.80	99.72%	98.90%	98.89%	179/181

**Table 11:** Repeated duplicate-group-aware validation using five repetitions of five-fold StratifiedGroupKFold. Identical 14-feature vectors were constrained to the same fold; values are mean +/– SD across 25 paired folds.

Model	OA, %	95% CI for OA, %	Weighted F1, %
RF	99.63 +/– 0.26	99.52–99.74	99.63 +/– 0.26
GBDT	99.29 +/– 0.46	99.10–99.48	99.29 +/– 0.46
RF-GBDT equal fusion	99.33 +/– 0.43	99.15–99.51	99.33 +/– 0.43

**Figure 9:**
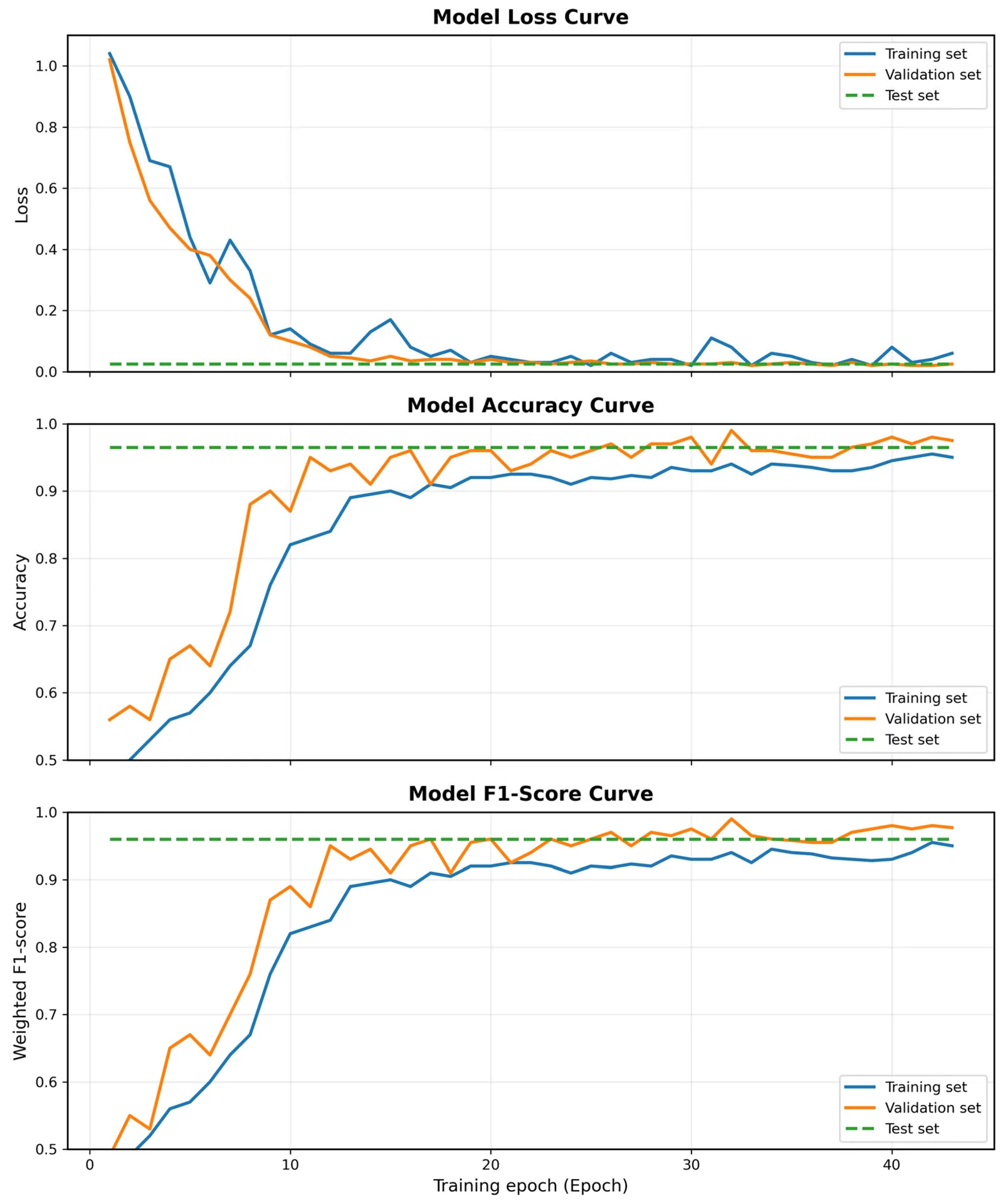
Training loss, accuracy, and weighted F1 trajectories for the improved deep branch under the random ROI-level train-validation split.

The training curves in Figure 9 show a smooth decrease in loss and stabilization of both training and validation metrics, indicating stable optimization. The repeated duplicate-group-aware evaluation of the tree branches provided a robustness assessment under stricter record separation.

**Figure 10:**
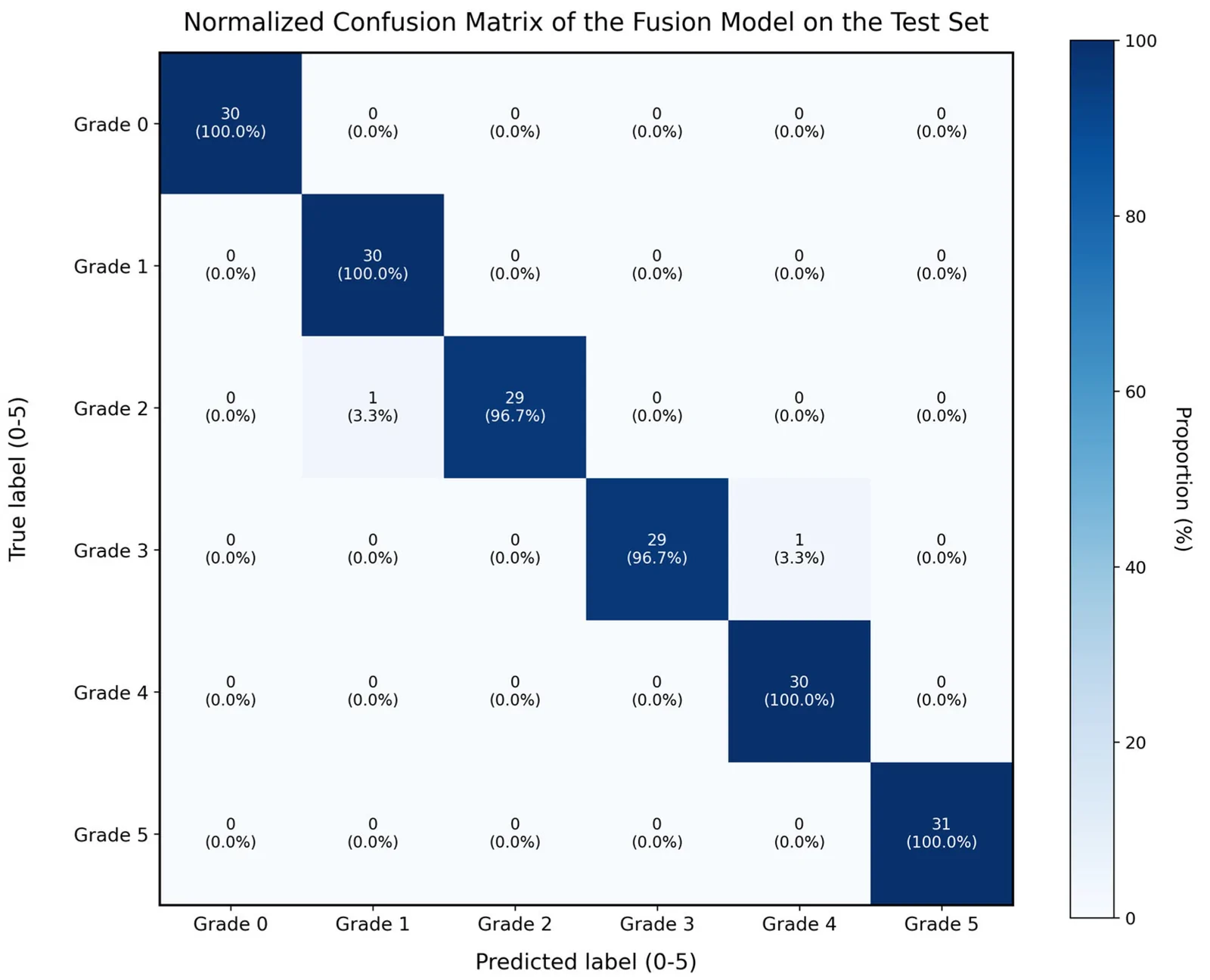
Confusion matrix of the 1DCNN-Transformer + RF + GBDT model on 181 test ROIs; of these, 179 were classified correctly, and the two errors occurred between adjacent severity classes.

The confusion matrix was strongly concentrated along the diagonal, with 179 of 181 test ROIs correctly classified. The two misclassifications occurred between adjacent severity classes (Figure 10).

The ablation results indicate that Inception, SE recalibration, and Focal Loss improved the deep branch, whereas RF and GBDT provided strong decision boundaries for the structured tabular inputs. Under repeated duplicate-group-aware validation, the tree-based models remained stable, with RF achieving the highest mean accuracy; RF-GBDT fusion did not statistically outperform RF.

### 3.4. Spatial Visualization of Rice Leaf Blast

For spatial deployment, the trained model was applied at the pixel level to UAV multispectral TIFF images. The workflow incorporated TIFF reading, pixel-level vegetation-index calculation, batch inference, and visualization while preserving the geographic metadata of the source images. Disease-severity predictions could therefore be mapped back to their field locations.

Spatial products were generated using the same 14-dimensional feature configuration employed for model development. Specifically, four original spectral bands and the 10 vegetation indices selected by Pearson correlation analysis were calculated for each valid canopy pixel. The resulting 14-feature vectors were arranged in the same order as the training data and processed in batches for pixel-level inference. This ensured consistency between ROI-level model development and field-scale spatial prediction.

For pixel-level deployment, image-derived feature vectors were assembled in a fixed order, processed in small batches to control memory use, and mapped to their original geographic coordinates. This procedure preserved image georeferencing while enabling continuous field-scale prediction.

**Figure 11:**
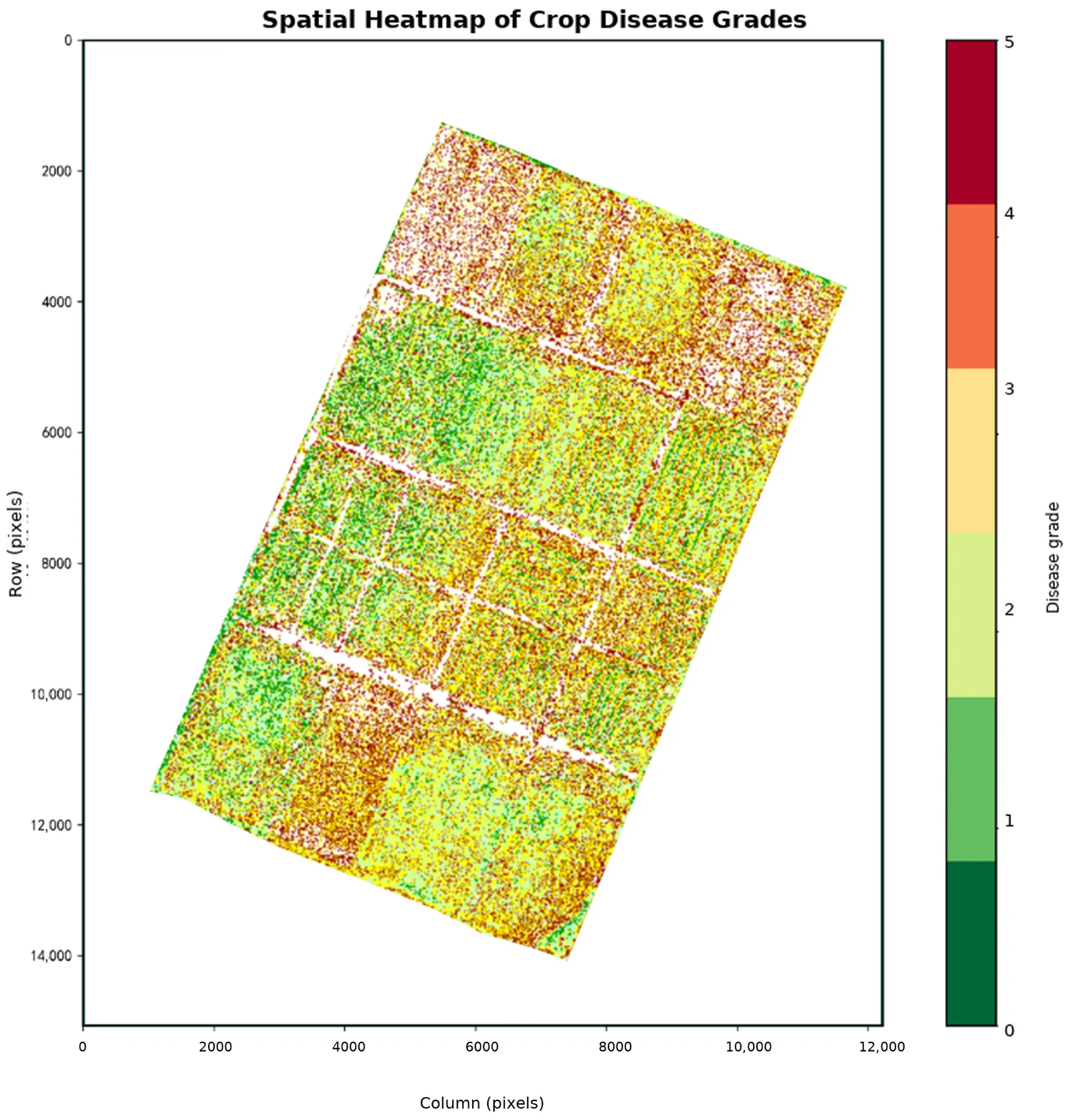
Spatial distribution of predicted rice leaf blast severity grades in the experimental field. Grades 0–5 are represented from dark green to dark red.

The two-dimensional severity matrix was visualized with a color map and saved as a georeferenced TIFF. Grade 0 was shown in dark green, grades 1–4 progressed from light green through yellow to light red, and grade 5 was shown in dark red (Figure 11). In this experimental field, predicted healthy areas were concentrated mainly in the central and eastern portions, whereas higher predicted severity occurred more frequently along the western and northern edges. The map provides a spatial summary of classifier outputs under the experimental acquisition conditions.

### 3.5. Generation of the Variable-Rate Application Prescription Map

The disease-severity map was used to construct a variable-rate prescription map. ArcGIS spatial tools vectorized the class map, extracted grade boundaries, and calculated mapped area proportions. In the experimental field, the mapped proportions for grades 0–5 were 37.84%, 16.20%, 8.92%, 14.32%, 13.24%, and 9.45%, respectively (Table 12). These proportions provide a compact quantitative summary of the predicted spatial disease pattern.

The prescription rule linked mapped severity classes to pesticide doses using local production practices and green-control recommendations [10]. Using 75% tricyclazole wettable powder as the reference, the nominal range was 20–30 g/mu with water for UAV spraying: class 0 received no pesticide, class 1 received 20 g/mu, classes 2–3 received 25 g/mu, and classes 4–5 received 30 g/mu (Table 13). These rule-based dose levels provide the operational input used to generate the prescription map.

Candidate aggregation grids of 6 m × 10 m, 12 m × 16 m, 12 m × 20 m, 16 m × 32 m, and 24 m × 40 m were compared against the severity map and field layout. Smaller cells retained greater spatial detail but fragmented the operation map, whereas larger cells simplified route execution but smoothed small disease patches. The 16 m × 32 m cell provided an operational compromise that aligned with the field geometry, produced 30 manageable units, and supported swath-by-swath route planning. Flight speed, nozzle configuration, spray width, flow rate, and boundary overlap can be specified according to the operating requirements of the selected UAV platform.

**Figure 12:**
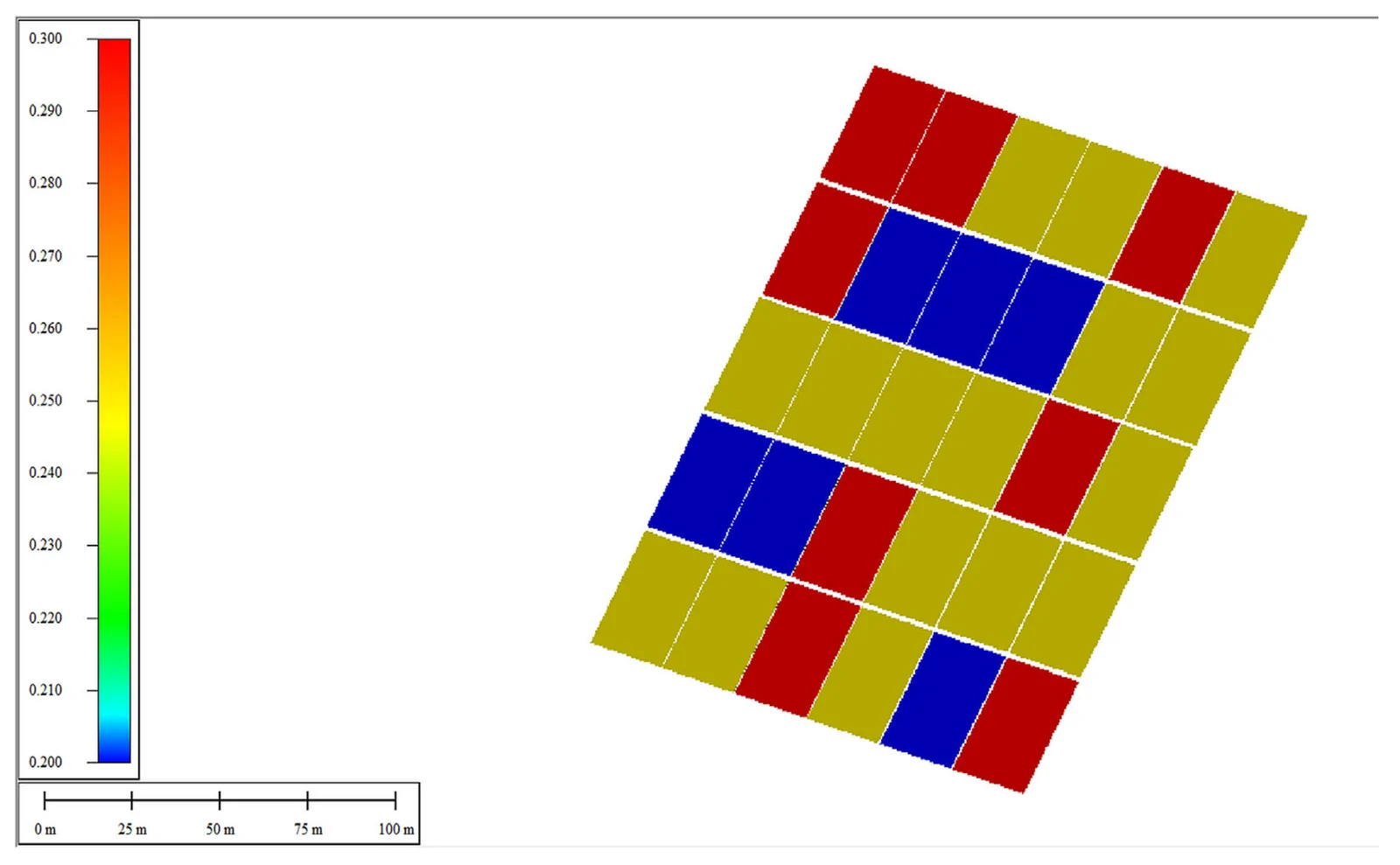
Variable-rate prescription map generated from the predicted severity distribution using 16 m × 32 m operation units.

Using the selected grid, the field was divided into 30 operation units, each assigned a nominal dose. Global Mapper converted the application-rate TIF into an elevation-grid format containing dose, geographic coordinates, and elevation. The file can be transferred to a DJI T60 controller through the DJI agricultural service platform or by SD card (Figure 12). The evaluation was limited to the digital workflow from severity mapping to generation of an operational prescription file; agronomic efficacy and economic performance were outside the scope of this remote-sensing and decision-support study.

## 4. Discussion

### 4.1. Effect of Complex Background Removal on Multispectral Feature Extraction

Complex paddy backgrounds introduce mixed pixels whose reflectance combines canopy, water, soil, ridges, and weeds. The present U-Net mask follows the encoder–decoder segmentation principle of Ronneberger et al. [22] and the rice-field extraction strategy reported by Cao et al. [23]. Its validation Dice coefficient of 93.36% indicates substantial mask-label overlap and supports canopy-focused feature extraction. Nevertheless, residual omission or commission errors at canopy boundaries can propagate into vegetation indices and class predictions. The Dice coefficient quantifies canopy-mask overlap and does not directly measure disease-severity classification performance.

### 4.2. Analysis of Vegetation-Index Screening Results

The vegetation-index associations have a plausible physiological basis. Visible and near-infrared reflectance respond to pigment content and canopy structure, while red-edge indices are sensitive to chlorophyll decline and stress. Rice leaf blast develops continuously: incremental lesion expansion and coalescence reduce chlorophyll, alter red and red-edge absorption, and modify near-infrared scattering. Canopy mixing within a 3 × 3-pixel ROI and the discretization of a continuous disease index can therefore produce spectral similarity between adjacent severity classes, consistent with the observed confusion between neighboring classes. The relationships of NDRE, SCCCI, OSAVI, GNDVI, RVI, GCI, and NDVI with severity are consistent with these physiological responses. Among the 20 candidate vegetation indices, the 10 indices showing stronger associations with disease severity were retained and combined with the four spectral bands. This 14-predictor feature set provided the input for subsequent severity classification and spatial mapping.

### 4.3. Performance Differences and Feature Complementarity Among Grading Models

The ablation results clarify the sources of the reported performance. InceptionTime demonstrates how parallel 1D kernels can capture patterns at multiple scales [29], and SE blocks recalibrate informative channels [30]. Focal Loss emphasizes difficult samples [31], which is relevant to adjacent severity boundaries even in a balanced dataset. For the 14-dimensional tabular input, however, RF [32] and GBDT [33] matched the rounded test performance of the full fusion. The principal empirical finding is therefore the strong performance of tree ensembles on this dataset, with the deep branch providing a complementary representation rather than a demonstrated gain in accuracy.

Comparisons with previous UAV-based rice blast studies should account for differences in task definition and validation design. Zhao et al. [20] evaluated early leaf blast detection using a disease-specific hyperspectral index and machine-learning models, whereas Liu et al. [21] used UAV hyperspectral imagery with a multi-scale attention Transformer. The present study employs a lower-dimensional four-band multispectral sensor and targets six-class canopy-level severity classification and spatial mapping. This lower-dimensional configuration may facilitate repeated field deployment, although it provides less spectral detail than hyperspectral imaging and makes rigorous spatial validation especially important. The 2026 comparison of UAV multispectral rice-disease mapping networks by Ghimire et al. [7] further underscores the value of standardized datasets and multiple segmentation metrics. Direct numerical comparisons should therefore be interpreted cautiously because the diseases, datasets, tasks, and data-partition strategies differ among studies.

### 4.4. Applicability Under Controlled Experimental Conditions

The high classification accuracy is consistent with several features of the experimental design. Artificial inoculation produced a controlled and well-spanned severity gradient; UAV acquisition and preprocessing were standardized across campaigns; 3 × 3-pixel averaging reduced single-pixel noise; and red-edge and near-infrared features captured physiologically relevant disease responses. The balanced six-class sampling design also prevented majority-class dominance. In repeated duplicate-group-aware validation, identical 14-feature records were kept within the same fold, yet mean accuracies remained above 99% for RF, GBDT, and their equal-probability fusion. This result indicates that exact duplicate records alone did not explain the high performance. The analysis does not establish spatial or plot-level independence; inference is therefore limited to discrimination within the controlled experimental site and acquisition protocol and is not extrapolated to unrelated regions.

### 4.5. Scope and Applicability

This study evaluated an integrated technical workflow for canopy extraction, six-class disease-severity modeling, spatial visualization, and prescription-file generation. The controlled inoculation experiment, single cultivar, and standardized acquisition protocol reduced environmental variability and facilitated evaluation of the spectral and modeling components. Figure 11 and Figure 12 show how class predictions can be translated into spatial decision-support products. Pesticide efficacy, yield response, and economic performance were not evaluated in the present study and require dedicated field validation.

## 5. Conclusions

This study developed an integrated UAV multispectral workflow for grading rice leaf blast severity under controlled field induction. Image registration, band synthesis, U-Net segmentation, and mask processing reduced interference from complex paddy backgrounds, and the U-Net achieved a validation Dice coefficient of 93.36%. The dataset comprised 1801 curated 3 × 3-pixel canopy ROIs, each represented by four spectral bands and 10 selected vegetation indices (14 predictors), and supported model training, evaluation, and spatial mapping.

The improved 1DCNN-Transformer combined multi-scale Inception convolution, SE recalibration, Focal Loss, RF, GBDT, and validation-based probability weighting. The hold-out evaluation yielded OA, Precision, Recall, and weighted F1 values of 98.90%, 98.93%, 98.90%, and 98.89%, respectively. Across five repetitions of five-fold duplicate-group-aware validation, RF, GBDT, and equal RF-GBDT fusion achieved mean accuracies of 99.63%, 99.29%, and 99.33%, respectively. These consistently high grouped-validation results support strong separability among the six severity classes within the experimental acquisition domain.

The classification outputs were translated into a spatial severity map and a 16 m × 32 m prescription map containing 30 operation units. These products establish a practical digital link between UAV-based disease assessment and field-scale decision support. The resulting workflow supports disease-severity classification, spatial mapping, and prescription-file generation; future field experiments are required to evaluate agronomic efficacy and economic benefits.

## Figures and Tables

**Table 1:** Spectral bands, center wavelengths/bandwidths, and effective pixel counts of the DJI Mavic 3 Multispectral sensor used at a 30 m flight height.

Sensor	Band/nm	Effective Pixels/Ten Thousand
Visible light	-	2000
Green	560 +/– 16	500
Red	650 +/– 16	500
Red edge	730 +/– 16	500
Near infrared	860 +/– 26	500

**Table 2:** Standard individual-leaf blast field ratings (0, 1, 3, 5, 7, and 9), representative values, and symptom descriptions used to calculate the plot-scale disease index.

Field Grade	Representative Value	Description
Level 0	0	No lesion on leaves
Level 1	1	Fewer than 5 lesions per leaf, with single-lesion length < 1 cm
Level 3	3	6–10 lesions per leaf, with some lesions > 1 cm
Level 5	5	11–25 lesions per leaf; some connected lesions; diseased area 10–25%
Level 7	7	26 or more lesions; connected lesions; diseased area 26–50%
Level 9	9	Connected lesions with diseased area > 50%, or fully withered leaves

**Table 3:** Conversion of plot-scale disease-index (DI) values into the six canopy severity classes (0–5) used for model training and mapping.

Grade	Severity	Disease Index
0	Healthy	DI = 0
1	Mild	0 < DI ≤ 5
2	Slightly mild	5.1 < DI ≤ 10
3	Moderate	10.1 < DI ≤ 20
4	Moderately severe	20.1 < DI ≤ 30
5	Severe	DI > 30

**Table 4:** Final U-Net segmentation configuration selected for 256 × 256-pixel patches; 140 annotated patches were used for training and 60 for validation.

Parameter	Setting
Input image size	256 px × 256 px
Optimizer	RMSprop
Initial learning rate	1 × 10^−5^
Learning-rate schedule	ReduceLROnPlateau
Loss function	Cross-entropy/BCE loss + Dice loss
Training mode	AMP
Epochs	300
Batch size	1

**Table 5:** U-Net canopy-segmentation result after 300 epochs, reported as validation Dice coefficient (*n* = 60 validation patches) and final training loss.

Metric	Validation Dice Coefficient	Training Loss
U-Net	93.36%	2.38%

**Table 6:** Number and proportion of ROI-level canopy samples across disease grades.

Disease Grade	Sample Number	Proportion
0	300	16.66%
1	300	16.66%
2	300	16.66%
3	300	16.66%
4	300	16.66%
5	301	16.71%

**Table 8:** Six modifications evaluated relative to the original 1DCNN-Transformer baseline, including their abbreviations and positions in the deep or ensemble workflow.

No.	Improvement	Abbreviation	Position
1	Multi-scale 1D Inception convolution branch	Inception 1D/Deep	CNN part of deep branch
2	SE feature recalibration attention	SE/Deep	Input features and fused features
3	Focal Loss	Focal/Deep	Training loss of deep branch
4	Random Forest ensemble branch	RF	Independent tabular classifier
5	Gradient Boosting Decision Trees branch	GBDT	Independent tabular classifier
6	Validation-set probability-weighted fusion	Fusion/Stacking	Probability fusion of Deep, RF, and GBDT

**Table 9:** Hold-out performance of the baseline, improved deep branch, and three-branch model on 181 records from the single 1801-record dataset.

Model	Test OA	Precision	Recall	F1	Correct Samples
Original 1DCNN-Transformer baseline	96.13%	96.28%	96.13%	96.13%	174/181
Improved deep branch	96.13%	96.46%	96.13%	96.19%	174/181
Deep + RF + GBDT fusion model	98.90%	98.93%	98.90%	98.89%	179/181

**Table 12:** Predicted area proportions of severity grades 0–5 in the experimental field, calculated from the model-generated spatial classification.

Disease Grade	0	1	2	3	4	5
Area proportion	37.84%	16.20%	8.92%	14.32%	13.24%	9.45%

**Table 13:** Rule-based pesticide doses and spray volumes assigned to mapped severity grades for prescription-map generation.

Disease Grade	0	1	2	3	4	5
Pesticide dose	0 g/mu	20 g/mu	25 g/mu	25 g/mu	30 g/mu	30 g/mu
Spray volume	0 L/mu	15 L/mu	18.75 L/mu	18.75 L/mu	22.5 L/mu	22.5 L/mu

## Data Availability

The original contributions presented in the study are included in the article. Further inquiries can be directed to the corresponding author.
